# Dry-Spun Neat Cellulose Nanofibril Filaments: Influence of Drying Temperature and Nanofibril Structure on Filament Properties

**DOI:** 10.3390/polym9090392

**Published:** 2017-08-25

**Authors:** Shokoofeh Ghasemi, Mehdi Tajvidi, Douglas W. Bousfield, Douglas J. Gardner, William M. Gramlich

**Affiliations:** 1 Laboratory of Renewable Nanomaterials, School of Forest Resources and Advanced Structures and Composites Center, University of Maine, Orono, ME 04469, USA; shokoofeh.ghasemi@maine.edu; 2 Department of Chemical and Biological Engineering, University of Maine, Orono, ME 04469, USA; dbousfield@umche.maine.edu; 3 School of Forest Resources and Advanced Structures and Composites Center, University of Maine, Orono, ME 04469, USA; douglasg@maine.edu; 4 Department of Chemistry, University of Maine, Orono, ME 04469, USA; william.gramlich@maine.edu

**Keywords:** cellulose nanofibrils, filaments, spinning, drying temperature

## Abstract

Cellulose nanofibrils (CNF) were spun into filaments directly from suspension without the aid of solvents. The influence of starting material properties and drying temperature on the properties of filaments produced from three different CNF suspensions was studied. Refiner-produced CNF was ground using a microgrinder at grinding times of 50 and 100 minutes. Filament spinning was performed using a syringe pump-heat gun setting at three drying temperatures of 210 °C, 320 °C and 430 °C. The structure of starting CNF materials was first evaluated using a combination of optical and atomic force microscopy (AFM) techniques. Surface free energy analysis and attenuated total reflectance—Fourier transform infrared spectroscopy (ATR–FTIR) were used to study changes in hydrophobicity due to grinding. Morphology of the filaments was studied using SEM micrographs. The influence of different drying temperatures and grinding times on mechanical properties of the CNF filaments were further investigated through tensile tests and results were compared using statistical analysis .It was observed that drying temperature did not significantly influence the tensile properties of the filaments while cellulose nanofiber suspension type (grinding time) had a significant influence and improved mechanical properties. FTIR results confirmed an increase in crystallinity index and decrease in hydroxyl group availability due to grinding.

## 1. Introduction

Most commercially available nanomaterials lack biodegradability or recyclability, thus have limited applications where recyclability and renewability are of interest. Cellulose nanofibrils (CNF), which are mechanically derived from wood pulp are widely regarded as materials that have the potential to help the declining pulp and paper industry by providing added value to low-demand wood pulp. These nanoscale fibrils offer an interesting package of physical and mechanical properties, making them potential candidates for a wide variety of applications to solve issues with current materials [1,2].

While conventional natural fibers such as hemp, kenaf and jute satisfy the bio-degradability requirements, they cannot compete with synthetic fibers in terms of performance. CNF has exceptional properties which are promising to fill the material property gap between natural fibers and technical materials [1,2]. Studies on the properties of different cellulose nanomaterials have resulted in the emergence of many new applications [3,4]. One of the key potential applications of the cellulose nanofibrils is the use of these materials in textile products as high-tech or conventional products. For these materials to be usable for most textile applications, they should first be produced in the form of textile filaments or yarns. As cellulose nanofibrils are naturally produced in the form of a low consistency slurry (about 97% water content), a method of producing filaments is applicable only if it can easily remove this large volume of water in the suspension while maintaining nanofibril alignment and avoiding agglomeration. 

The different methods proposed for the production of cellulose nanofibril filaments can be classified into two categories: wet and dry-spinning. Wet spinning refers to processes in which the spun filaments enter a coagulation bath before being dried. On the other hand, dry-spinning methods do not use such a bath. Hooshmand et al. (2015) [5] used a dry-spinning method to produce CNF filaments. They used a capillary rheometer to extrude the CNFs. They also studied the influence of different CNF concentrations (8, 10 and 12 wt %) and spinning speeds (72, 144 and 216 mm/s) on the mechanical properties of the resulted filaments as well as nanofibril orientation. It was revealed that the best mechanical properties belonged to the filaments produced using the lowest solids content and highest spinning rate. Iwamoto et al. (2011) [6] used wet-spinning of TEMPO (2,2,6,6-Tetramethylpiperidin-1-yl)oxyl)-mediated wood pulp and tunicate cellulose and studied the influence of different spinning rates on the alignment and strength of the spun filaments. They used spinning rates of 0.1–100 m/min. It was reported that as the spinning rate increased, the mechanical properties of the filaments increased as a consequence of the increase in orientation. The authors reported that the highest modulus belonged to filaments spun from nanofibrils spun at 100 m/min.

Walther et al. (2011) [7] proposed a method for wet spinning renewable cellulose nano-fibrils. Their CNF production process was similar to TEMPO -mediated oxidation that was followed by homogenization using a microfluidizer. They used a syringe for wet extrusion of the CNF hydrogels in an organic solvent. The resultant macro-fibers were reported to have comparatively high mechanical properties. Lundhal et al. (2016) [8] used TEMPO-mediated CNF and CNF hydrogels for the production of the filaments and studied water interaction, alignment and strength of the resulted filaments. They used CNF hydrogels with solid contents of 2 wt %, 5 wt % and 7 wt % and TEMPO oxidized CNF (TOCNF) with 2 wt % solid content and reported that the highest tensile modulus among filaments produced belonged to CNF 2 wt %. Hakanson et al. [9] proposed a method for the production of filaments from low-concentration CNF suspension (3 g L^−1^) in water using hydrodynamic alignment with a dispersion-gel transition to help align fibrils in the filament structure. They used a fixed core flow rate using different sheath flow rates and different sheath NaCl concentrations. Reported mechanical properties of the filaments using the proposed method showed maximum mechanical properties for the filaments spun using the highest NaCl concentration (100 mM) and the highest acceleration rate (1.15, sheath rate/core rate).

While a number of research groups have focused on CNF filament production and properties as influenced by spinning rate, solids content, draw ratio and alignment, no reports are available to provide information on the effects of the structure of the starting CNF material and drying conditions (drying rate) on the properties of the resultant filaments. In the present work, we have proposed a spinning assembly based on dry-spinning and studied the influence of different drying temperatures and cellulose nanofibril types on the properties of the continuous filaments. 

## 2. Materials and Methods 

The original CNF suspension used for the production of filaments had a solids content of 3 wt % and was supplied by the University of Maine’s Process Development Center. This CNF material is produced by mechanically refining softwood Kraft pulp to a 95% fines content without any chemical treatments. This product was used as unground CNF without further grinding. Two other grades of CNF suspensions were produced in the lab from the original CNF by grinding it in a micro-grinder (Masuko Sangyo, Model: MKCA6-2/, Kawaguchi, Japan) for 50 and 100 min. Throughout this paper, CNF 50G and CNF 100G refer to CNF that was ground for 50 and 100 min, respectively. The original (unground) CNF is designated as UG in this paper. The grinding procedure was carried out at 1 wt % solids content and suspensions used for spinning procedure were all at 1 wt % solids content.

**Figure 1:**
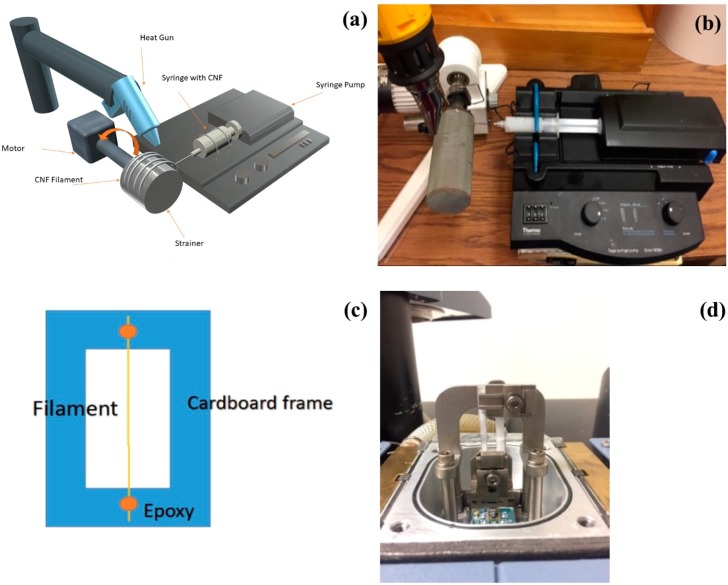
Filament spinning method and tensile testing (**a**) schematic representation of the spinning assembly; (**b**) photo of spinning assembly and heat gun; (**c**) schematic presentation of the sample tabbing for tensile test and (**d**) sample in dynamic mechanical analyzer (DMA) machine.

Filament production was undertaken using a dry spinning/drying setting. A schematic of the spinning and drying assembly is presented in Figure 1a and the spinning assembly and heat gun position are shown in Figure 1b. The syringe spinning was followed by a take-up roller which was heated by a heat gun mounted at 10 cm distance. To help the extruded structures keep their circular cross-section, the surface of the take-up roller was sprayed with cooking oil to reduce surface tension as suggested by Shen et al., 2016 [10].

The spinning speed was constant during spinning and was set at 11 m/min. Take-up speed was also set constant for 12 m/min during filament production. Spinning was done using a 30 mL syringe and a blunt needle with 1.48 mm diameter. CNF filaments were produced using three different types of cellulose nanofibril suspensions (unground, 50 min. ground and 100 min. ground) and were dried under three different heating protocols of 210 °C, 320 °C and 430 °C. It is important to note that these temperatures are temperatures set at the heat gun and the actual temperature on the filament surface would never reach these values because of the presence of liquid water. The highest surface temperature measured on filaments was 92 °C for the 430 °C filament at drying point. Air dried samples were also made at room temperature as a reference. Average filament diameters after drying were 0.15 mm for air-dried filaments, 0.17 mm for those dried at 210 °C, 0.16 mm for 320 °C and 0.17 mm for the filaments dried at 430 °C, because of the CNF shrinkage during drying. Table 1 shows different sample combinations and codings.

To study the influence of grinding procedure on the properties of CNF suspensions, different studies were conducted on CNF films. These included surface properties and fibril size and fibril size distribution as well as determination of crystallinity index.

Microscopic methods were used to study the influence of grinding on the cellulose nanofibril suspensions. Very dilute suspensions of each CNF type were poured on a microscope glass slide and microscopic images were obtained from dried CNF films formed on the slide. An AmScope microscope (Model ME520TA, Irvine, CA, USA) was used to image the samples from which microscale particle size and particle size distributions were determined through image analysis by analyzing at least 100 fibrils of each CNF type.

Atomic force microscopy (AFM) was used to image the surface of films produced from a dilute suspension of each CNF type formed on a glass slide cover. A countertop ezAFM atomic force microscope (Nanomagnetics Instruments, Oxford, UK) was used to evaluate the fibrils at nanoscale and for the comparison of fibril size distribution of different CNF suspensions. At least 100 fibrils of each CNF type were evaluated.

To study the influence of grinding on the surface properties of dried CNF materials, films were produced from 1 wt % suspension of each CNF type through solution-casting at room temperature. A Mobile Surface Analyzer (MSA, KRÜSS GmbH, Hamburg, Germany) was used to measure surface properties of the films. Contact angles were measured using two liquids of water and diiodomethane. Surface free energy (determined using the OWRK model [11]), as well as polar and disperse parts of the surface free energy were calculated using the surface analyzer and accompanying software.

To study if the grinding process brought about any changes in the chemical structure and hydroxyl group availability of the CNF suspensions and to determine the crystallinity index for comparing crystallinity of the CNF suspensions [12], films of each CNF type were dried in an oven at 100 °C overnight, and ATR–FTIR spectra of the surface were obtained using a Spectrum Two IR spectrometer (Perkin Elmer, Waltham, MA, USA).

After understanding the property differences between different CNF suspensions, a number of characterization methods were used to characterize filaments produced from these CNFs and dried under different drying temperatures.

Scanning electron microscopy was used to study surface properties, microscale alignment and cross-section circularity of the filaments. A desktop Hitachi scanning electron microscope (SEM), TM 3000 (Hitachi, Ltd., Tokyo, Japan) was used for SEM imaging. No sputter coating is required for imaging using the TM 3000. Micrographs of surface and cross-section of the filaments produced from different cellulose nanofibril suspensions and using different drying temperature were obtained.

## 3. Results

### 3.1. Fibril Size and Fibril Size Distribution

**Figure 2:**
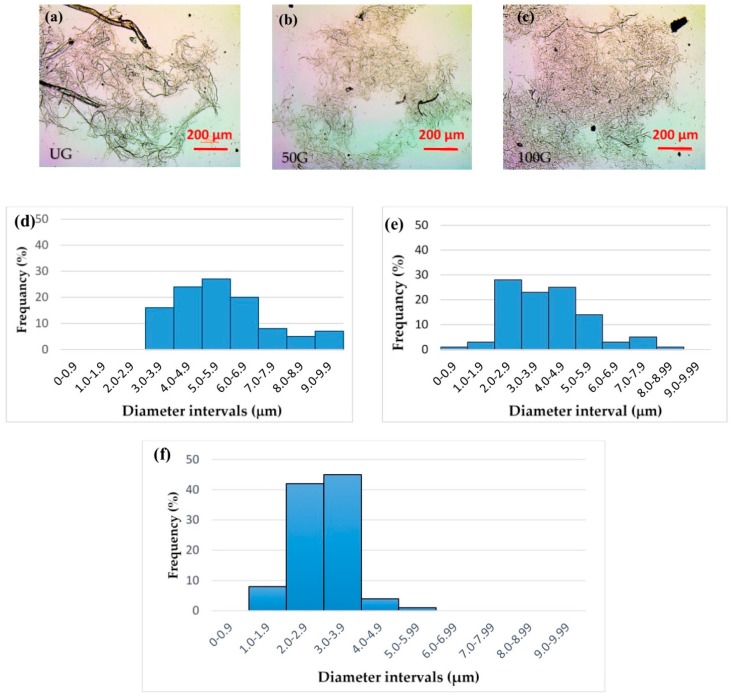
Microscopic images of the very dilute suspensions of Cellulose nanofibril (CNF) after drying and particle size distributions: (**a**) CNF UG; (**b**) CNF 50G; (**c**) CNF 100G. Histograms of fibril size distribution for different CNF suspensions: (**d**) CNF UG; (**e**) CNF 50G; (**f**) CNF 100G.

As shown in Figure 2, grinding resulted in a considerable decrease in fibril size and fibril size distribution in the suspensions. As the optical microscopic method (Figure 2a–c) provides information on the fibrils on the micron-size scale, the influence of grinding on fibril size on the micron scale along with fibril size distribution for each suspension is reported here. To evaluate fibril size changes on the nano size-scale, AFM imaging was used. This practice has been previously reported by our research group and provides a better understanding of fibril size and fibril size distribution by combining micro- and nano-scale characterization methods [13].

To study tensile properties of the filaments, tensile tests were done using a dynamic mechanical analyzer (DMA) in static mode. A DMA Q800 was used to run tensile tests on filaments. Tensile testing was carried out according to ASTM D3822. 10 specimens from each group of filaments were used for tensile tests. Specimens were tabbed according to ASTM D3822 (Figure 1c) with 25.4 mm gauge length and were tested using a controlled force method (Figure 1d). Ramp force of 2 N/min was applied on the samples until failure and tensile properties including tensile modulus and tensile strength were calculated.

### 3.2. AFM Characterization

**Figure 3:**
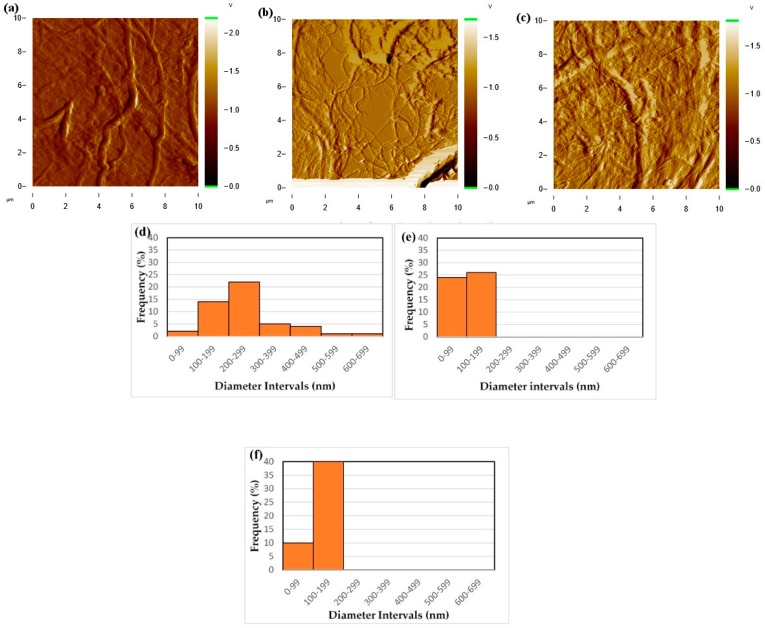
Atomic force microscopy (AFM) imagery of the CNF films: (**a**) CNF UG; (**b**) CNF 50G; (**c**) CNF 100G. Histogram of fibril size distributions for different CNF suspensions using AFM micrographs: (**d**) CNF UG; (**e**) CNF 50G; (**f**) CNF 100G.

AFM in tapping mode was used to scan the surfaces of the samples made from drying a very dilute drop of each CNF suspension on a glass surface. These images are presented in Figure 3a–c. Studies on the line surface roughness of different CNF suspensions was done using the control software of the AFM device and average roughness of 0.06 µm was reported for unground CNF while it was 0.03 µm for CNF 50G and 0.05 µm for CNF 100G.

As shown in Figure 3d–f, grinding resulted in more fibrils with nano-scale diameters compared to the unground sample. Similar to the optical microscopy method, fibril size and size distribution were measured from AFM images (Figure 3a–c). As shown in Figure 3, grinding decreased fibril size on the nanoscale; fibril size distribution also became narrower by grinding.

### 3.3. Surface Properties of CNF Films

Results obtained from surface analysis of films produced from different CNF suspensions were compared and reported in Table 2.

As shown in Table 2, grinding resulted in an increase in water contact angle and decrease in surface free energy of the films. The polar portion of surface free energy showed a considerable reduction suggesting the less hydrophilic nature of highly ground CNF material and potentially a decrease in the available hydroxyl groups. Previous studies have mentioned parameters influencing contact angle and surface energy of cellulose structures. Surface roughness and chemical composition are two of the most important factors influencing the contact angle of cellulose films and papers [14,15,16]. These factors were evaluated here. 

### 3.4. ATR–FTIR Results

**Figure 4:**
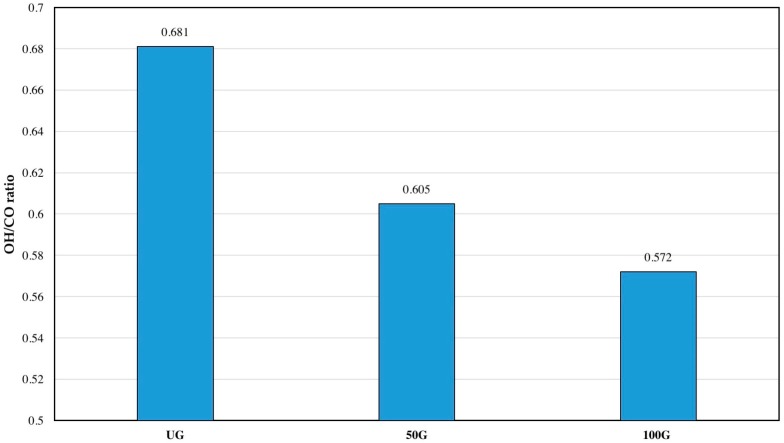
OH/CO ratio of different CNF suspension films as obtained from attenuated total reflectance–Fourier transform infrared spectroscopy (ATR–FTIR).

Attenuated total reflectance–Fourier transform infrared spectroscopy (ATR–FTIR) was used to study the influence of grinding on chemical structure, in particular, accessibility of hydroxyl groups on the surface of the films as a proxy for the surface properties of CNF filaments. Hydroxyl groups are responsible for hydrogen bonding in cellulose, and the structure of cellulose is highly influenced by the hydrogen bonds made by OH groups [17]. Absorbance values were obtained and the ratio between -OH content (represented as area under the peak at 3400 cm^−1^) and -CO content (represented as area under the peak at 1050 cm^−1^), which is also referred to as the hydrogen bond intensity (HBI) [18,19] was calculated and compared for the three types of films. Results are presented in Figure 4 where the O–H/C–O ratio showed a decrease as a result of grinding CNF suspensions. Crystallinity index was also calculated using the ratio of the absorbance value at 1427 cm^−1^ and 895 cm^−1^. These two peaks are attributed to CH_2_ bending and deformation of anomeric CH [12,20]. The crystallinity index showed to increase from 0.91 for the UG film to 1.12 for the 50G film and 1.23 for the 100G film, which is an indicator of increase in crystallinity by grinding.

### 3.5. Morphology of the Filaments

**Figure 5:**
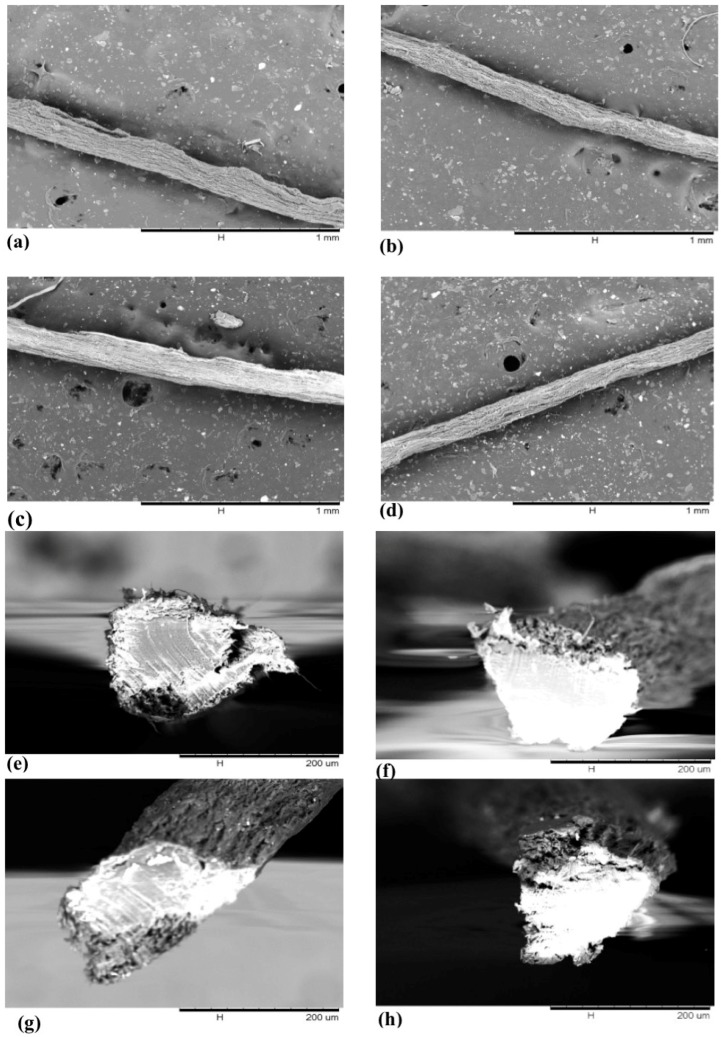
SEM micrographs from the surface of filaments-dried under different temperatures: (**a**) CNF 100G air-dried; (**b**) CNF 100G 210 °C; (**c**) CNF 100G 320 °C; (**d**) CNF 100G 430 °C. SEM micrograph of cross-sections of filaments dried at different temperatures: (**e**) CNF 100G air-dried; (**f**) CNF 100G 210 °C; (**g**) CNF 100G 320 °C; (**h**) CNF 100G 430 °C.

SEM micrographs of the filaments dried under different drying temperatures are presented in Figure 5. These micrographs were evaluated to study the influence of drying temperature and CNF grinding on the morphological properties of the filaments.

As shown in Figure 5a–d, drying temperature did not seem to make a difference in filament structure. SEM micrographs were also obtained from cross-sections of the filaments dried under different temperatures to evaluate the influence of drying rate on the structure of the filaments. These micrographs were obtained for all three types of CNF suspensions at four different drying temperatures. As the general morphology was similar for different CNF suspensions, SEM micrographs of the 100G CNF formulation are presented in Figure 5 for brevity. As seen in Figure 5e–h, the cross-section of the filaments were almost the same with relatively acceptable circularity despite the fact that very dilute suspensions were spun.

### 3.6. Tensile Properties of Filaments

**Figure 6:**
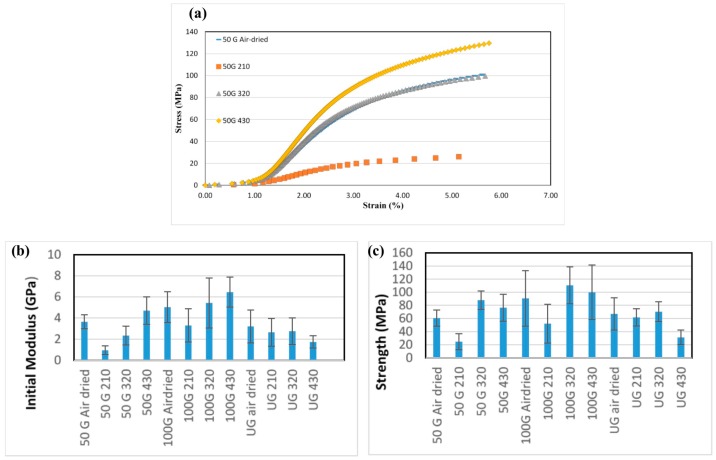
(**a**)Typical stress/strain curves of filaments made from the 50G sample dried at different temperatures. Results of a tensile tests of the filaments: (**b**) Young’s modulus or initial modulus of the filaments; (**c**) strength of the filaments.

Figure 6a shows a typical comparative stress/strain graph for the 50G filaments produced using different drying temperatures. As shown in Figure 6a, samples of filaments that were dried at 210 °C exhibited the weakest mechanical properties. As diameter and density measurements did not confirm a consistent trend, we were not able to understand why this particular sample was weaker than others. Figure 6b,c present all the results of the tensile tests on the samples. Filaments produced from CNF 100G showed the best mechanical properties of both initial modulus and strength. Initial modulus is the slope of the first linear part of the stress-strain curve also called Young’s modulus or elastic modulus. In addition, samples dried at 430 °C exhibited acceptable mechanical properties compared with air-dried samples. It is notable that samples dried at 430 °C were heated for less than 1 min, while those that were air-dried were left to dry for 24 h. The significance of the differences between the tensile properties of filaments produced from different CNF types and at different drying temperatures was evaluated using statistical analysis. These will be discussed in the following section.

## 4. Discussion

The influence of drying temperature and starting CNF material structure on the properties of filaments produced from CNF has not been studied before. As CNF is naturally produced as a suspension containing about 97% water, the drying procedure is a time-consuming, expensive and crucial step for the production of any material from cellulose nanofibrils, in particular CNF filaments. This emphasizes the importance of optimizing drying conditions to obtain the highest mechanical properties in the shortest time.

Studies on the CNF suspensions produced using different grinding times showed that grinding resulted in a significant decrease in the size of the fibrils and also decreased variability in the fibril size. AFM images supported the finding that fibrils decreased in size as grinding time increased. AFM images provided more information about the appearance of more fibrils with nanoscale width because of disentanglement during grinding procedure. ATR–FTIR results also confirmed increase in the crystallinity of the CNF suspensions by grinding.

**Figure 7:**
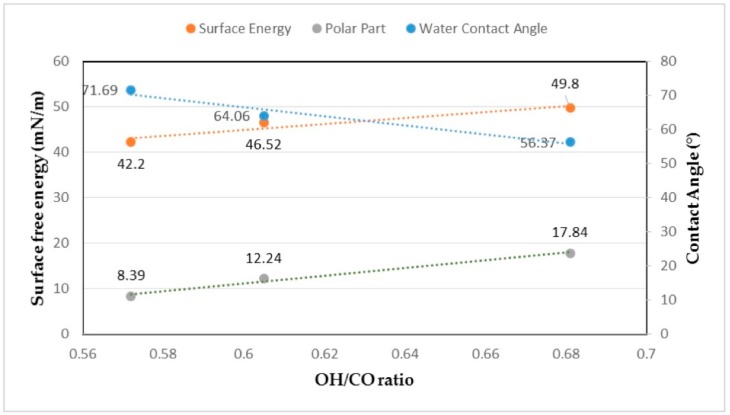
Influence of OH/CO ratio on the contact angle, surface free energy and polar part of the surface free energy of the films (the values of OH/CO for different CNF suspensions are: CNF UG = 0.681, CNF 50G = 0.605, CNF 100G = 0.572).

Surface analysis was conducted to see if grinding has an influence on the surface properties of the films as they are expected to be highly hydrophilic because of the presence of hydroxyl groups. The results showed a notable decrease in the surface free energy of the films produced from ground CNF and an increase in the water contact angle of the surface of the films. To study if these differences are a result of chemical changes, indicating more internal interactions of the hydroxyl groups, or they are a result of change in the surface roughness, ATR–FTIR results were obtained. These results showed that the ratio of O–H groups to C–O groups decreased by grinding CNF suspensions. Surface analysis and ATR–FTIR results were complementary indicating a decrease in available hydroxyl groups on the surface perhaps as a result of increased internal hydrogen bonding after grinding. According to Wenzel’s equation, for a droplet on a hydrophilic surface, contact angle will also decrease with an increase in surface roughness [14,15,16]. As mentioned, AFM results of surface roughness showed a roughness value of 0.06 µm for CNF UG, while the value of surface roughness was 0.03 µm for CNF 50G and 0.05 µm for CNF 100G. On the other hand, a decrease in the ratio of the OH/CO group will also result in a decrease in available hydroxyl groups and thus decrease in active groups that can bond with water. Figure 7 shows the influence of these chemical parameters on surface properties of the films. By increasing OH/CO (an increase in available hydroxyl groups) water contact angle decreased, while surface free energy and the polar component of surface free energy increased. It seems that both chemical and physical parameters are influencing surface properties of CNF films. For a better understanding of changes in chemical and physical properties due to grinding, hydroxyl number determination on super-critically dried CNF samples can be helpful.

**Figure 8:**
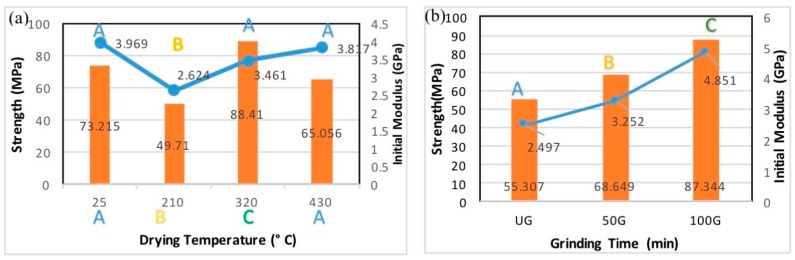
(**a**) Influence of drying temperature on initial modulus and strength of filaments classified according to drying temperature (each bar represents average value for filaments produced from different CNF types dried at the temperature listed); (**b**) Influence of CNF grinding time on initial modulus and strength of filaments.

Figure 8a shows the comparison of strength and Young’s modulus of filaments dried at different temperatures. Two-way analysis of variance (ANOVA) was used to study differences between different groups of samples. Results of Duncan’s multiple range test are also shown on the graphs in Figure 8. Values with the common letter are not significantly different at a 95% confidence level. The difference between samples dried at different drying temperatures was only significant between filaments dried at 210 °C and other samples. It is notable that the difference between Young’s modulus values of samples dried at 430 °C and air-dried samples was not statistically significant. The difference was more noticeable for strength values but, still not significant between samples dried under 430 °C and the ones that were air-dried. The ANOVA test on samples produced using different CNF suspensions showed significant differences between different groups, and Duncan’s test classified samples into three different subsets. Filaments produced from CNF suspension ground for 100 min had the highest mechanical properties. The increase in the mechanical properties of filaments is the result of both physical and chemical parameters. It can be attributed to the mechanical interlocking of fibrils, the decrease in roughness by grinding, and hydrogen bonding of the more available hydroxyl groups which will result in less available hydroxyl groups on the surface and will support the results of surface analysis. Further evaluations of the role of each parameter will need more in-depth analysis of the hydroxyl groups and crystalline structure. These will remain interesting areas to explore in future work.

Crystallinity and fibril orientation are known to influence the mechanical properties of CNF filaments significantly. Crystallinity index calculated for different CNF suspensions confirmed the increase previously reported by others [4] due to grinding. The best mechanical properties among filaments produced using different CNF suspensions and different drying temperatures belonged to the filaments produced using CNF 100G dried at 430 °C. Filaments produced from CNF 100G at 430 °C had a Young’s modulus of 6.5 GPa and strength of 100 MPa. This can be attributed to increase in the crystallinity by grinding CNF. In this work, filaments were produced without any drawing with constant spinning rate. Thus, no difference between fibril’s orientation in the filament structure was expected. This was confirmed by polarized light microscopy where no considerable color change was observed when filaments were rotated between cross-polars. It is expected that spinning rate and drawing would result in significant increase in the mechanical properties as they will increase fibril orientation.

Results of tensile tests undertaken by Hooshmand et al. [5] showed that the highest mechanical properties corresponded to the filaments produced at the lowest solids content and highest spinning rate; the highest Young’s modulus reported was 12.6 GPa and the highest strength was 222 MPa which belonged to the filaments produced at 8 wt % solids content and 216 mm/s spinning rate. Iwamoto [6] used wet spinning with different spinning rates and reported that the best mechanical properties were obtained for filaments spun using the highest spinning rate approaching 23 GPa Young’s modulus and 321 MPa strength. Walther et al. [21] proposed a new CNF production method along with syringe spinning in a coagulation bath, and reported mechanical properties of 22.5 GPa Young’s modulus and strength of 275 MPa. Lundhal et al. also used wet spinning in a coagulation bath, for CNF and TOCNF in the range of 2–7 wt % solids content, and reported that the highest mechanical properties between CNF samples belonged to the filaments produced using CNF2 wt % which was 15.5 GPa of Young’s modulus and a strength value of 326 MPa. The filament produced using TOCNF was reported to have Young’s modulus of 21.3 GPa and 297 MPa strength. Hakanson et al. [9] proposed a method for the production of filaments from low-concentration CNF suspensions in water using hydrodynamic alignment with a dispersion-gel transition. This method resulted in filaments with mechanical properties of 18 GPa Young’s modulus and 490 MPa strength—the highest ever reported.

The difference between mechanical properties of our filaments and filaments produced using other methods is thought to be related to the spinning method and spinning rate as well as the CNF type and drawing process, which can increase fibril orientation in the filaments and result in structures with higher mechanical properties. Nonetheless, these comparisons are important as they provide a better insight into potential property improvements through raw material selection and process optimization. 

## 5. Conclusions

In this work, we have studied the influence of starting CNF type (as affected by grinding time) and drying temperature on the properties of filaments produced by dry spinning of cellulose nanofibril suspensions. Optical microscopic and AFM images showed a decrease in the fibril size of the suspensions with grinding more uniformity in fibril size distribution after grinding and decrease in surface roughness. ATR–FTIR results also confirmed increase in the crystallinity of the CNF with grinding. These changes positively contributed to better mechanical properties of the produced filaments, potentially by increasing mechanical interlocking and hydrogen bonding. Surface analysis showed a decrease in surface energy, and an increase in water contact angle, of films with grinding, which can be attributed to a decrease in available hydroxyl groups. ATR–FTIR results showed a decrease in the ratio of OH/CO groups by grinding, which can also be a result of a decrease in available hydroxyl groups by an increase in internal hydrogen bonds involved in the hornification phenomenon. Studies on the properties of filaments produced using the presented spinning method showed uniformity in the cross-section of the filaments despite varying drying temperatures. SEM micrographs showed that cross-sections of the filaments had acceptable circularity. Studies on the tensile properties of filaments showed that those dried at 430 °C were not significantly different from those that were air-dried. In addition, CNF type had a significant influence on the properties of the filaments, and filaments produced using CNF ground for 100 min proved to have the highest mechanical properties. We conclude that the drying section of dry spinning for CNF filaments can be carried out at higher temperatures for a shorter period, saving time and increasing production rates. A more uniformly distributed particle size distribution with more nano-scale fractions can also improve mechanical properties.

## Figures and Tables

**Table 1:** Different sample combinations and coding.

Cellilose Nanofibril (CNF) Type	Air Dried-25 °C	210 °C (400 °F)	320 °C (600 °F)	430 °C (800 °F)
Unground (original CNF)	UG-Air-dried	UG-210	UG-320	UG-430
50-min ground CNF	50G-Air-dried	50G-210	50G-320	50G-430
100-min ground CNF	100G-Air-dried	100G-210	100G-320	100G-430

**Table 2:** Surface properties of films made from different CNF suspensions at room temperature.

CNF Type	Water Contact Angle [°]	Diiodomethane Contact Angle [°]	Surfacefree Energy [mN/m] (OWRK Model)	Disperse Portion [mN/m]	Polar Portion [mN/m]
UG	56.37 (9.94)	54.10 (2.01)	49.80 (7.39)	31.96 (1.14)	17.84 (6.24)
50G	64.06 (7.28)	50.00 (5.33)	46.52 (7.21)	34.27 (2.97)	12.24 (4.24)
100G	71.69 (14.67)	50.84 (8.11)	42.20 (11.88)	33.80 (4.55)	8.39 (7.33)
